# A Quality Improvement Project to Address Inequalities in Access to Admission to a Mother and Baby Unit in Kent Across a 3-Year Period

**DOI:** 10.1192/bjo.2025.10415

**Published:** 2025-06-20

**Authors:** Sophie Warner, Isobel Thomas, Bosky Nair, Chidi Nwosu

**Affiliations:** 1 Maidstone and Tunbridge Wells Hospital, Maidstone, United Kingdom; 2 Kent & Medway NHS Trust, Maidstone, United Kingdom; 3 Kent & Medway NHS Trust, Dartford, United Kingdom

## Abstract

**Aims:** Rosewood Mother and Baby Unit (MBU) provides inpatient psychiatric care to women with severe mental illness in Kent, Surrey & Sussex (KSS) in UK. Analysis of admission data to the MBU in 2022 highlighted inequalities in the admissions process. A quality improvement project was undertaken to improve equity of access for women irrespective of their ethnicity, location or age.

**Methods:** We collated data on admissions to Rosewood MBU, including demographics, origin of referrals, diagnosis, length of stay, parity, Mental Health Act status, previous MBU admissions and safeguarding concerns. In the 2024 cohort, additionally, we looked at suspected or confirmed neurodevelopmental disorders such as ASD or ADHD and deprivation decile to establish the socio-economic status of admitted patients.

The project group undertook consultations with referrers, inpatient/community teams and other stakeholders to understand barriers to referrals from various counties, for women of black and ethnic minority backgrounds and under-18s. We implemented measures such as improving ethnicity recording, building partnerships with community groups to raise awareness and build trust, offering appropriate training for staff working with young and ethnically diverse mothers, providing easy access to information about referrals pathway and role of MBU to referrers and families.

**Results:** In 2022, the duration of admission was less than 2 months for 64% of patients, which increased to 77% in 2023 and 70% in 2024. Psychotic illness was the most common diagnosis for patients admitted in 2022 and 2024, while anxiety-related illness was most common in 2023.

In 2022, 10% of admitted patients were of black, Asian or mixed backgrounds, which increased to 33% in 2023 and 36% in 2024. In 2023 and 2024, there were 2 referrals and 1 admission of women under the age of 18, compared with no referrals in 2022.

In 2024, mean age of mothers admitted was 30.6 years with a range between 19 and 40 years. 18% of patients had suspected or confirmed diagnosis of Autism Spectrum Disorder or Attention Deficit Hyperactivity Disorder. In the same year, 39% of admissions had lower socio-economic status with deprivation decile between 1–4.

**Conclusion:** Overall, this project demonstrates a positive trend with improved access for under-18s and women of black, ethnic minority and mixed ethnic population groups across the 3-year span. Further work is needed to improve access for women living in more deprived areas and to recognise and support women with neurodevelopmental disorders in the perinatal period.

